# Supplementary material for: Relationship between landslide susceptibility and social lag in Mexico City: The case of the west periphery
Source: PLoS One. 2026 Jan 9;21(1):e0340639. doi: 10.1371/journal.pone.0340639 (PMC12788685; doi:10.1371/journal.pone.0340639)
Supplement: Attachment — Submitted filename: Relationship between.docx [file pone.0340639.s001.docx]

**Relationship between landslide susceptibility and social lag in Mexico City: the Case of the West Periphery**

**To Editor**

The manuscript titled "Relationship between landslide susceptibility and social lag in Mexico City: the Case of the West Periphery" presents an interesting approach by integrating landslide susceptibility analysis with socio-economic factors. The study has potential, but the methodological weaknesses, poor validation, contradictions, and missing scientific rigor necessitate a **major revision**. The authors need to rethink their modeling approach, strengthen their validation, and resolve inconsistencies before the manuscript can be reconsidered for publication. However, the paper contains several technical mistakes, scientific flaws, methodological gaps, and missing key components that need major revisions before it can be considered for publication.

**To Author:**

1. The authors claim that they chose the Frequency Ratio (FR) method due to its simplicity, interpretability, and its presence in previous literature. However, this is not a sufficient justification. Given the availability of more robust machine-learning techniques for susceptibility mapping, such as Random Forests (RF), Support Vector Machines (SVM), or Deep Learning (CNN), the manuscript should justify why FR was the best choice for this study.
2. The authors acknowledge that machine learning techniques are more advanced but dismiss them without testing or benchmarking them against FR. A comparative analysis between FR and ML methods should be included to validate this choice.
3. The study attempts to establish a relationship between social lag and landslide susceptibility using multinomial logistic regression. However:
   i. The model's pseudo R² value is extremely low (0.007352), indicating an extremely weak fit.
   ii. The p-values for most coefficients are non-significant, implying that the hypothesis of an association between landslide susceptibility and social lag is not supported.
   iii. The authors acknowledge this limitation but do not propose a corrective measure, such as feature selection, interaction effects, or alternative modeling approaches.
4. The authors use Huber regression to study individual social lag components, citing outlier robustness as the main reason. However:
   i. The rationale for choosing Huber regression over other robust techniques (e.g., Quantile Regression, Ridge/Lasso Regression) is missing.
   ii. Collinearity issues among social lag variables are not addressed, potentially leading to misleading results.
   iii. The paper lacks model diagnostics, such as residual plots or variance inflation factor (VIF) analysis, to confirm the robustness of Huber regression.
5. The study lacks an independent validation dataset of past landslides to assess the accuracy of the generated susceptibility map.
6. It is crucial to calculate performance metrics, such as the Area Under the Curve (AUC) of the Receiver Operating Characteristic (ROC) curve, for landslide susceptibility models.
7. Mexico City has various landslide hazard maps published by government agencies or research institutions.
8. The study should compare the proposed susceptibility map with official landslide hazard maps to assess its reliability.
9. The results section contradicts the initial hypothesis. The authors hypothesize that social lag should correlate with landslide susceptibility but later find that areas with high landslide susceptibility tend to have lower social lag.
10. This contradiction is not well explained, nor is an alternative hypothesis proposed.
11. The study groups AGEBs into Low, Medium, and High social lag categories but ignores the Very High and Very Low categories that might contain critical insights.
12. The omission of these categories raises concerns about selection bias in the analysis.
13. The study refers to flow-like landslides without precisely defining them in the Mexican geographical context. Are these debris flows, mudflows, or rock avalanches?
14. The classification of landslide types should be explicitly stated, preferably with references to recognized classification systems such as Cruden and Varnes (1996) or Hungr et al. (2014).
15. The study uses DEM data, land cover maps, and social indicators, but it does not explain:
    i. The spatial resolution of all datasets and whether resampling was performed.
    ii. How missing data was handled in the social lag variables.
    iii. Whether the temporal mismatch between datasets (e.g., 2007 DEM, 2020 social data) could introduce bias.
16. The susceptibility model does not include uncertainty quantification. Given that landslide modeling is inherently probabilistic, the study should report confidence intervals for its estimates.
17. Several statements (e.g., social vulnerability theories, hydrological influence on landslides) lack citations from peer-reviewed sources.
