# Supplementary material for: Relationship between landslide susceptibility and social lag in Mexico City: The case of the west periphery
Source: PLoS One. 2026 Jan 9;21(1):e0340639. doi: 10.1371/journal.pone.0340639 (PMC12788685; doi:10.1371/journal.pone.0340639)
Supplement: Attachment — Submitted filename: Response to Reviewers.docx [file pone.0340639.s002.docx]

**Dear Editor and Reviewers,**

Thank you so very much for taking the time to make such a comprehensive review of our work. Thanks to it, we have come with a total refurbishment of the article: we have addressed each and every comment you kindly made, and also we made additions that go beyond the kind recommendations you made. We prepared a letter which address each point raised by the Editor and both of the reviewers plus the additions we deemed appropriate to fill the gaps you mentioned.

**The revised Tracked changes version contains colored in Blue the new additions, and in yellow the parts which we took from the final version.**

**Editorial Comments.**

The manuscript “Relationship between landslide susceptibility and social lag in Mexico City: The Case of the West Periphery” presents an interesting topic that integrates landslide susceptibility analysis with socio-economic factors. However, based on the two detailed reviews, there are significant methodological concerns that must be addressed before the manuscript can be reconsidered.
Key Issues to Address in the Revision:

**1. Justification of the Frequency Ratio (FR) Method:
o Reviewer 1 suggests that the use of FR needs stronger justification, especially given the availability of more advanced machine learning techniques (e.g., Random Forest, SVM, CNN).
o A comparative analysis should be conducted between FR and an ML-based model to demonstrate its effectiveness.**

Dear Editor and Reviewers, you are absolutely right, the justification presented to use the Frequency Ratio method is not enough. Consequently, we have now estimated 13 models to generate the susceptibility map, including but not limited to the ones suggested by the reviewers and editors e.g. Random Forest, SVM, Deep Learning and more. For each model we included the ROC curve and the AUC Value. This process allowed us to determine that Frequency Ratio was not the best model although it did a good job. We then changed Frequency Ratio by an XG Boost, which had the best performance according to the aforementioned metrics. The champion model was an XGBoost which is now used to generate the susceptibility map.

**2. Weak Statistical Model for Social Lag Analysis:
o The multinomial logistic regression model shows an extremely weak fit (pseudo R² = 0.007352), making the relationship between social lag and landslide susceptibility statistically weak.
o Alternative modeling techniques (e.g., feature selection, interaction terms, or different regression models) should be considered.**

Dear Editor, thank you so much for this comment. You are so right, we have now moved entirely to a new and different approach, using SHAP values derived from a Random Forest model to visually determine the association magnitude and direction. Additionally, we used this model to be able to calculate an R-squared metric and have a performance metric about how reliable this analysis is. We anticipate that we got 80% explained variance which is a decent statistic and which then provides credibility to our SHAP values. Furthermore, for each partition we made we estimated Copula Models to provide a robust magnitude and direction metric for the association between Landslide Susceptibility and Social Lag.

**3. Validation of the Landslide Susceptibility Model:**
o **The lack of independent validation data is a major concern. It is crucial to include:
 A validation dataset of past landslides to assess accuracy.
 Performance metrics such as AUC-ROC curves to validate the predictive strength of the model.**

You are absolutely right. In this new version, we include validation metrics for each susceptibility map created by 13 models. We include ROC and AUC and throughout them, it was possible to discriminate models, and to finally select the XGBoost.

**4. Contradictions in Results and Hypothesis:**
**o The manuscript hypothesizes that higher social lag should correlate with higher landslide susceptibility, but findings show the opposite trend.
o A clearer explanation of this contradiction is required. If the hypothesis is incorrect, it should be revised with alternative theoretical support.**

This revised version not only includes new models to create susceptibility maps, it also includes new ways to assess the relationship we are studying: Landslide Susceptibility vs Social Lag, in this new version we found significant relations between the study variables provided that we partition the dataset by deciles and using a Changepoint algorithm. Both partitions allowed us to study this relationship with more granularity, and it helped to avoid noise that comes from studying a large heterogeneous area such as the western periphery of Mexico City. With this new approach, it was possible to find a positive association in many deciles and segments partitioned by the Changepoint algorithm.

**5. Choice of Huber Regression and Lack of Diagnostics:**
**o The rationale for using Huber regression should be justified over other robust regression techniques (e.g., Quantile Regression, Ridge Regression).
o The study lacks model diagnostics (e.g., residual analysis, VIF, or multicollinearity tests) to verify the robustness of Huber regression.**

Again, you are absolutely right. In this new version we changed the models and we included performance metrics. As stated before, we first used random forest to derive SHAP Values as an exploratory way to visually study the magnitude and direction of the relationship, for this model we included R-squared metric for each of the segements. Secondly, we used Copulas as a robust association method and include the Loglikelihood coefficient.

**6. Missing Data Issues and Temporal Bias in Data Sources:**
**o There is no explanation of how missing values in social lag variables were handled.
o The temporal mismatch between datasets (e.g., 2007 DEM vs. 2020 social data) could introduce bias.**

Thank you very much for this suggestion. You are absolutely right, in this new version the whole Data Sources section was re-written and we included every relevant detail that is required, including the missing values, an explanation about the temporal mismatch -we anticipate that the dataset was not so mismatched, the maps used were 2017 not 2007 as we initially claimed, it was an error on our side and we apologize. Additionally, we include data about the re-projection and methods used, we are sure this new section is totally illustrative about data’s metadata.

**7. Comparison with Official Landslide Hazard Maps:
o Given that Mexico City already has existing landslide hazard maps, the manuscript should compare its susceptibility map with official hazard maps to assess its reliability.**

Thank you very much. This is obviously a valid concern. We found the official CENAPRED’s landslide susceptibility map. It is a categorized version which means it colors risk-zones according to a pre-defined range. Since this is the official version, we compared our susceptibility map with this one to provide a better assessment.

**8. Lack of Uncertainty Quantification:
o Landslide susceptibility models are probabilistic in nature. The study should provide confidence intervals for its estimates**.

We absolutely agree with this regard. To this end, we did the following “As a way to quantify uncertainty in the susceptibility map, we achieved this through a bootstrap resampling framework, which generates an ensemble of predictions to capture variability arising from sampling and model training processes. Specifically, we did 50 bootstrap iterations, wherein each iteration involves resampling the subsampled dataset with replacement to form a bootstrapped training set, followed by fitting an XGBoost classifier and predicting susceptibility probabilities across the cleaned feature matrix. The collection of these predictions, enables the derivation of ensemble statistics: the maximum (susceptibility_max) and minimum (susceptibility_min) values which represent the upper and lower bounds of prediction variability. These Max and Min maps make it straightforward to show the whole range of variability within the output.”

**9. Inadequate Literature Citations and Reference Formatting Issues:**
o **Reviewer 2 highlights improper references, missing citations from high-impact peer-reviewed sources, and unstructured reference formatting.
o The literature review should incorporate recent and relevant citations, including key works on landslide susceptibility modeling.**

Thank you very much for this comment. We revised our references and modified the main text and reference list accordingly; we added new ones and replaced the conflicting ones. The final version includes all of these changes.

**10. Data Resolution and Spatial Analysis Issues:**
**• There is no explanation of how datasets with different spatial resolutions (e.g., Landsat, MODIS, and DEM data) were standardized.
• Image enhancement or fusion techniques may be required to ensure data consistency.**

You are absolutely right. We re-write the vast majority of the Data Sources section to clarify exactly what we did about the spatial resolution, how it all fell into the same, comparable standard, the methods used, and the final resolution. We summarized all this information into a table at the end of this Data Sources section.

**11. Missing Discussion:**
• **Sensitivity analysis should be performed to quantify the impact of different variables on landslide susceptibility.
• The manuscript lacks a proper discussion of computational efficiency, feature importance, and the limitations of the proposed method.**

Thank you for this comment. In the new version we added three dimensions of sensitivity analysis: for the susceptibility map model we added XGBoost Feature Importance. Also, we added the Information Gain Ratio, and the GeoDetector q-statistic. For the association between SLI and Landslide susceptibility, in one setting, we calculated dependence between each of the variables with which SLI was made and Landslide Susceptibility.

Additionally, we included the limitations and efficiency in the XGBoost section since this was the model we used: “XGBoost complexity depends on the number of trees, features, tree-depth and data. It's parallel logic translates even complicated calculations into relatively simple threads. Although efficient, its limitations lie in that it has many hyperparameters to estimate, making it resource-intensive; it also tends to overfit data even with regularization; finally, it tends to be classified as black-box since the modeler is not fully aware of the model's inner calculations~\cite{bib72}”.

**12. Both reviewers express concerns about language clarity, grammatical errors, and ambiguous phrasing. A thorough English language revision is necessary to ensure the manuscript is clear, coherent, and professionally structured.**

I know, and I apologize about that! This version was reviewed several times, exclusively looking for grammar mistakes. We also tried to make sentences more concise.

###################################################################

Additional Editor Comments:

The manuscript “Relationship between landslide susceptibility and social lag in Mexico City: The Case of the West Periphery” presents an interesting topic that integrates landslide susceptibility analysis with socio-economic factors. However, based on the two detailed reviews, there are significant methodological concerns that must be addressed before the manuscript can be reconsidered.

Key Issues to Address in the Revision:

**1. Justification of the Frequency Ratio (FR) Method:
o Reviewer 1 suggests that the use of FR needs stronger justification, especially given the availability of more advanced machine learning techniques (e.g., Random Forest, SVM, CNN).
o A comparative analysis should be conducted between FR and an ML-based model to demonstrate its effectiveness.**

Sir, thank you so very much for your comment. We agree that the use of FR method needs stronger justification. Yet, we did something bigger: as suggested, we calculated several other models-12 in total- to generate the susceptibility map, including many Machine Learning models, and we compared them using the ROC/AUC plot and metric. The champion model was an XGBoost, and the justification for using it now is that it has the highest AUC.

**2. Weak Statistical Model for Social Lag Analysis:
o The multinomial logistic regression model shows an extremely weak fit (pseudo R² = 0.007352), making the relationship between social lag and landslide susceptibility statistically weak.
o Alternative modeling techniques (e.g., feature selection, interaction terms, or different regression models) should be considered.**

This is an absolutely valid concern. To completely solve it we recalculated everything by using new association methods which are SHAP values derived from a random forest, and COPULAS, which are excellent at modeling non-linear associations. Furthermore, to delve deeper into our dataset, we partitioned it into deciles and also by using a Changepoint partitioning algorithm. For each of the resulting segments, we applied the SHAP and COPULA Analysis resulting in many significant relationships. Additionally, to assess how valid was the application of the method, we used the r-squared derived from the random forest as a fitting metric. En each of the segments, that metric rounded 80%, which is a decent fit.

**3. Validation of the Landslide Susceptibility Model:
o The lack of independent validation data is a major concern. It is crucial to include:
 A validation dataset of past landslides to assess accuracy.
 Performance metrics such as AUC-ROC curves to validate the predictive strength of the model.**

Absolutely true, thank you for the comment. In this new version we included ROC-AUC plots and metrics for each of the models we used to create the landslide susceptibility map.

**4. Contradictions in Results and Hypothesis:
o The manuscript hypothesizes that higher social lag should correlate with higher landslide susceptibility, but findings show the opposite trend.
o A clearer explanation of this contradiction is required. If the hypothesis is incorrect, it should be revised with alternative theoretical support.**

Thank you so much again, and thanks for the patience of delving deeper into this point of our work. Yes, absolutely right, nevertheless we re-calculated the whole relationship with different methods and partitioning the dataset into deciles, with changepoints -and also doing it with the whole dataset. This new modeling approach did result into meaningful relationships which does not contradict the hypothesis. Thank you very much for the comment, and this limitation has been superseded.

**5. Choice of Huber Regression and Lack of Diagnostics:
o The rationale for using Huber regression should be justified over other robust regression techniques (e.g., Quantile Regression, Ridge Regression).
o The study lacks model diagnostics (e.g., residual analysis, VIF, or multicollinearity tests) to verify the robustness of Huber regression.**

Thank you for your valuable feedback. We agree that our initial use of Huber regression was a limitation, particularly regarding model diagnostics. To address this, we have replaced the Huber regression with a revised approach using SHAP values and Copula models. This change was made to enhance model interpretability and provide a more robust analysis. SHAP values directly address the lack of diagnostics by explaining the contribution of each feature to the model's output.

Regarding multicollinearity, we have maintained the exact covariates used by CONEVAL to ensure our findings are directly reproducible and comparable with the official Mexican Social Lag Index, which is a key objective of our study. This decision justifies including potentially collinear variables.

**6. Missing Data Issues and Temporal Bias in Data Sources**:
**o There is no explanation of how missing values in social lag variables were handled.
o The temporal mismatch between datasets (e.g., 2007 DEM vs. 2020 social data) could introduce bias.**

Thank you. Actually, it was a mistake on our side since the dataset dates 2017 except for the Land Cover and CONEVAL social lag, which dates 2020. Nevertheless, we address this issue in the reformulated version of the Data Sources section. Furthermore, we made explicit what are the missing data code and how they were managed in the Data Sources section:

“Invalid no-data values, are excluded using a common mask, and any remaining missing values in predictor variables are imputed with their mean across valid observations, though this approach assumes random missingness and may overlook spatial correlations. As stated, we used subsampling to reduce computational demands by limiting the dataset to a predefined maximum number of pixels, using stratified sampling to maintain class balance between landslide and non-landslide instances, thus mitigating potential bias without introducing new missing data.”

**7. Comparison with Official Landslide Hazard Maps:
o Given that Mexico City already has existing landslide hazard maps, the manuscript should compare its susceptibility map with official hazard maps to assess its reliability.**

Totally agree. This revised version includes a comparison with CENAPRED’s official map. CENAPRED is Mexico´s official disaster prevention agency, and we make the comparison with its categorical map, which is the only one they have publicly available.

**8. Lack of Uncertainty Quantification:
o Landslide susceptibility models are probabilistic in nature. The study should provide confidence intervals for its estimates.**

Thank you very much for this comment. We totally agree with you and we now include an UQ framework as follows: “We absolutely agree with this regard. To this end, we did the following “As a way to quantify uncertainty in the susceptibility map, we achieved this through a bootstrap resampling framework, which generates an ensemble of predictions to capture variability arising from sampling and model training processes. Specifically, we did 50 bootstrap iterations, wherein each iteration involves resampling the subsampled dataset with replacement to form a bootstrapped training set, followed by fitting an XGBoost classifier and predicting susceptibility probabilities across the cleaned feature matrix. The collection of these predictions, enables the derivation of ensemble statistics: the maximum (susceptibility_max) and minimum (susceptibility_min) values which represent the upper and lower bounds of prediction variability. ”

**9. Inadequate Literature Citations and Reference Formatting Issues:**
**o Reviewer 2 highlights improper references, missing citations from high-impact peer-reviewed sources, and unstructured reference formatting.
o The literature review should incorporate recent and relevant citations, including key works on landslide susceptibility modeling.**

Thank you very much for bringing this issue. The new version includes new references, and we also replaced the conflicting older references, thank you.

10. **Data Resolution and Spatial Analysis Issues:
• There is no explanation of how datasets with different spatial resolutions (e.g., Landsat, MODIS, and DEM data) were standardized.
• Image enhancement or fusion techniques may be required to ensure data consistency.**

We almost completely re-writte this whole Data Sources section to completely address this concern. The new version includes a very detailed description of the resolution, resampling and an explanation about the data resolution apparent mismatch, we addressed every concern raised within this section.

11**. Missing Discussion:**
**• Sensitivity analysis should be performed to quantify the impact of different variables on landslide susceptibility.
• The manuscript lacks a proper discussion of computational efficiency, feature importance, and the limitations of the proposed method.**

We are deeply thankful about bringing this concern. As stated before, in the new version we added three dimensions of sensitivity analysis: for the susceptibility map model we added XGBoost Feature Importance. Also, we added the Information Gain Ratio, and the GeoDetector q-statistic. For the association between SLI and Landslide susceptibility, in one setting, we calculated dependence between each of the variables with which SLI was made and Landslide Susceptibility.

Additionally, we included the limitations and efficiency in the XGBoost section since this was the model we used: “XGBoost complexity depends on the number of trees, features, tree-depth and data. It's parallel logic translates even complicated calculations into relatively simple threads. Although efficient, its limitations lie in that it has many hyperparameters to estimate, making it resource-intensive; it also tends to overfit data even with regularization; finally, it tends to be classified as black-box since the modeler is not fully aware of the model's inner calculations~\cite{bib72}”.

**12. Both reviewers express concerns about language clarity, grammatical errors, and ambiguous phrasing. A thorough English language revision is necessary to ensure the manuscript is clear, coherent, and professionally structured.**

Thank you very much for this comment. We proofread the final version several times to address this issue and to make this final version as best as possible in terms of language formality.

[Note: HTML markup is below. Please do not edit.]

Reviewers' comments:

Reviewer's Responses to Questions

**Comments to the Author**

1. Is the manuscript technically sound, and do the data support the conclusions?

The manuscript must describe a technically sound piece of scientific research with data that supports the conclusions. Experiments must have been conducted rigorously, with appropriate controls, replication, and sample sizes. The conclusions must be drawn appropriately based on the data presented.

Reviewer #1: Partly

Reviewer #2: No

2. Has the statistical analysis been performed appropriately and rigorously?

Reviewer #1: Yes

Reviewer #2: No

3. Have the authors made all data underlying the findings in their manuscript fully available?

The [PLOS Data policy](http://track.editorialmanager.com/CL0/http:%2F%2Fwww.plosone.org%2Fstatic%2Fpolicies.action%23sharing/1/010f019587b87f70-c58336c5-a7bf-4874-9306-46227e717f6d-000000/35_96JRYjzWL0enChmsUYvGKCyL6bioo3QygSYnSNjM=201) requires authors to make all data underlying the findings described in their manuscript fully available without restriction, with rare exception (please refer to the Data Availability Statement in the manuscript PDF file). The data should be provided as part of the manuscript or its supporting information, or deposited to a public repository. For example, in addition to summary statistics, the data points behind means, medians and variance measures should be available. If there are restrictions on publicly sharing data—e.g. participant privacy or use of data from a third party—those must be specified.

Reviewer #1: Yes

Reviewer #2: No

4. Is the manuscript presented in an intelligible fashion and written in standard English?

PLOS ONE does not copyedit accepted manuscripts, so the language in submitted articles must be clear, correct, and unambiguous. Any typographical or grammatical errors should be corrected at revision, so please note any specific errors here.

Reviewer #1: No

Reviewer #2: No

**Reviewer #2: A super simple straightforward application of FR for landslide hazard in a disorganized and unstructured draft.

English proficiency stands beyond quality due to frequent flaws, vague statements, linguistic flaws…**

Thank you for your comment. We have addressed this issue by proofreading and correcting every grammatical error found in the text. Hope the new version is sufficiently improved in terms of language.

**Improper references and commercial weblinks while solid publications are missing. Using a dissertation for FR from 2018 while this method has many solid references, ….**

Thank you very much for this comment. YoWe have added new references, we have changed the conflicting old references to make the new version solid.

**The next critical issue is the perquisite use of unified pixel size, and therefore it cannot be functional for extracted attributes from different imageries like Landsat, Modis, and … with different resolutions incorporated with raster data.

Therefore, based on the used datasets, it can only be acceptable when the inputs don’t change, else in the case of modified images (in pixel or grids) it will no longer match and hence, the integrity cannot be verified. In addition, the overlaying the raster and vector with different resolutions and geospatially distribution requires image enhance processing, or image fusion Technique. Novel approach can be found at** [**https://www.sciencedirect.com/science/article/abs/pii/S0341816219303674**](https://www.sciencedirect.com/science/article/abs/pii/S0341816219303674)**,** [**https://doi.org/10.1155/2023/9429505**](https://doi.org/10.1155/2023/9429505)**, …**

Thank you very much for this issue. We have re-written the entire Data Sources section to make explicit every detail with respect to the data sources, including resampling methods, projection, final resolution, etc.

**Without any doubt, REJECTED conclusion. 1) pretty long and unjustified, 2) it obviously is not the place for citation**

Thank you very much for your comment. The new version has a revised shorter conclusion wich only addresses the central points in a straightforward way.

**Overall,
1. Lack of novelty is concrete and beyond taking time for discussion**

Thank you very much for your point of view. We respectfully disagree with your perspective since there are not too many works that look for relationships between landslide susceptibility and social lag. In the new version we included new techniques to find an association between landslide susceptibility and social lag along dataset partitioning techniques. These techniques, which are Copula models and SHAP values allowed us to find meaninfgfull relationships in important segments of the dataset.

**2. Doesn’t provide proper analyzed research gaps, taxonomy, and meaningful guidelines in the domain.**

We acknowledge this concern and have revised the introduction and discussion to explicitly map existing studies into a clear taxonomy (physical-only, social-only, and integrated approaches). Within this framework, we identify the research gap our work addresses: the lack of reproducible, explainable models that jointly assess landslide susceptibility and social lag at the urban periphery. We also provide methodological guidelines, emphasizing data harmonization, explainable ML (SHAP), and robust validation.

**3. In comparison with advanced approaches For example,** <https://www.sciencedirect.com/science/article/abs/pii/S0341816219303674>, <https://link.springer.com/article/10.1007/s10346-019-01299-0>, <https://link.springer.com/article/10.1007/s100640050066>, <https://www.tandfonline.com/doi/abs/10.1080/02723646.2021.1978372>, <https://www.sciencedirect.com/science/article/pii/S2590056022000202>, <https://www.tandfonline.com/doi/abs/10.1080/01431161.2019.1672904>, … **it is uncompetitive.**

Thank you for pointing us to these advanced methods. We agree they represent strong state-of-the-art lines of work. Our goal in this paper is different: rather than proposing a new algorithm, we provide an interpretable, policy-oriented framework that links landslide susceptibility to social lag using

1. copula-based dependence analysis,
2. SHAP explanations, and
3. changepoint/decile partitioning to expose spatially heterogeneous regimes.

This combination is, to our knowledge, uncommon in the Mexico City context and is designed to be reproducible with widely available data and transparent for risk-management decisions. To address your concern and improve comparability, the revision includes (or will include in the next round):

- Stronger baselines and fair evaluation.
- We report XGBoost/LightGBM/Random Forest with spatial block cross-validation, AUC and PR-AUC.
- Direct comparison to “advanced” families.

We add a results subsection discussing the cited approaches (deep nets, kernel/SVM, hybrid remote-sensing pipelines), clarifying data needs (e.g., very high-resolution DEM/imagery, InSAR), computational cost, and transferability. Where feasible with our data, we include an additional benchmark (SVM with RBF kernel) and report metrics side-by-side.

What our approach adds beyond accuracy.

- Copulas: quantification of dependence strength and direction by segments/deciles (including tail-sensitive diagnostics).
- SHAP: local/global interpretability that consistently elevates educational deprivation and basic-infrastructure deficits, making the results auditable by stakeholders.
- Partitioning (changepoints/deciles): reveals regimes where high SLI coincides with high susceptibility versus resilient, self-contained communities—insights that black-box models often do not make explicit.
- Transparency and reproducibility.

In sum, we position the paper not as an algorithmic competitor to the most advanced pipelines, but as a complementary, interpretable framework that connects physical susceptibility with social vulnerability and yields actionable diagnostics. We believe these additions address the competitiveness concern while preserving the paper’s primary contribution.

**4. Lack of data analysis/data visualization in considering the selected attributes are opaque**

Thank you again for your comment. We regret that the older version did not include this. Nevertheless, the new version makes this explicit, we provide explicit visualization of each of the inputs used -DEM, Slope, Aspect, etc- along with the way each of the input was chosen and pre-processed (e.g. resampled) to become a valuable input for our analysis. Again, we are sorry that this was not done from the beginning.

**5. Lack of considering the effect of subsurface spatial soil/rock type distributions** (<https://link.springer.com/article/10.1007/s10064-018-1400-9>, <https://www.sciencedirect.com/science/article/abs/pii/S0169555X16306419>, <https://link.springer.com/article/10.1007/s10346-013-0409-1>, <https://www.sciencedirect.com/science/article/abs/pii/S0013795224002655>, <https://www.mdpi.com/2220-9964/10/5/341> ...), considering the clay sensitivity and its effect in landslide (<https://www.sciencedirect.com/science/article/pii/S0037073817303019>; <https://www.sciencedirect.com/science/article/abs/pii/S0013795215000411>, <https://www.sciencedirect.com/science/article/pii/S0341816222002752> …), spatial analysis of the collected data and involved uncertainty of the post processing geo-model (<https://link.springer.com/article/10.1007/s00366-023-01852-5>, …

Thank you for your kind comment, it would be greater for another paper to include subsurface distribution. Yet this input is not necessarily required in other papers already published by PlosONE, for instance:

<https://journals.plos.org/plosone/article?id=10.1371/journal.pone.0292897>

<https://journals.plos.org/plosone/article?id=10.1371/journal.pone.0245668>

Furthermore, the new version include a bootstrap resampling framework that generates ensemble predictions to model variability from sampling and training. These predictions made it possible to generate ensemble statistics: maximum /minimum susceptibility maps.

**6. You have different attributes. The sensitivity analysis and predictability SHOULD be carried out via weight database of the optimum model to show the importance of each selected features (Search for updating the neural network models using different sensitivity analysis methods, sensitivity analysis for neural networks, novel feature selection using sensitivity analysis…)**

Thank you very much. You are absolutely right in this issue. The new version includes Feature Importance, Information gain, and q-statistic, which we believe will totally fill this gap.

**7. Lack of any convincing and documented Discussion cannot be neglected. Solid comparison with other approaches and scholars/visualized results in compare with other scholars/computational time and cost based on the system and model used/technical limitations/uncertainty quantifications comparing with advanced automated predictive deep learning models..., impact of bias of the used data on the results, noise strategy removal, …**

Thank you very much for your concern. The new version includes a totally re-written discussion which addresses the central points of the results, providing explanations. We also compared our results with those of the official Mexican Agency for Disaster Prevention (CENAPRED), they generated a categorized susceptibility map which we included into our new version.Additionally we include the model (XGBoost) technical limitations, Uncertainty Quantification and Imputation methods, when those were necessary.

**8. Trivially ill-formatted reference list**

Thank you very much for your concern. We have addressed this issue and the new work contains new reference and we have removed the old conflicting references.
