# Supplementary material for: Relationship between landslide susceptibility and social lag in Mexico City: The case of the west periphery
Source: PLoS One. 2026 Jan 9;21(1):e0340639. doi: 10.1371/journal.pone.0340639 (PMC12788685; doi:10.1371/journal.pone.0340639)
Supplement: Attachment — Submitted filename: Plos one review.docx [file pone.0340639.s003.docx]

This revised manuscript is now a strong contribution to both landslide susceptibility modeling and urban vulnerability assessment. The authors have addressed the vast majority of methodological and presentation concerns raised by reviewers, positioning the study as a reproducible, policy-relevant framework for combined physical-social risk analysis in complex urban regions. Minor additional improvements to language and clarity are advisable, but on technical and scientific grounds, the manuscript now meets the standards of *PLOS ONE*, but I recommend the manuscript after addressing the below queries:

1. The computational framework—partitioning by decile and changepoint—represents a methodological advance suitable for broader application in social-ecological risk mapping.
2. Adding quantitative examples of the improvement in predictive accuracy from the switch to XGBoost and the impact of Copula/SHAP integration on policy inferences may further strengthen the manuscript’s claims.
3. Regular comparison with international examples or an even more explicit outline of how results were audited for reproducibility would be valuable for readers seeking to transfer methods to new contexts.
