# Supplementary material for: Relationship between landslide susceptibility and social lag in Mexico City: The case of the west periphery
Source: PLoS One. 2026 Jan 9;21(1):e0340639. doi: 10.1371/journal.pone.0340639 (PMC12788685; doi:10.1371/journal.pone.0340639)
Supplement: Attachment — Submitted filename: Response_to_Reviewers_auresp_2.docx [file pone.0340639.s004.docx]

**Dear Editor and Reviewers,**

Thank you very much for your kind comments and useful reviews in the last round. In what follows, we will provide a detailed in-line numbered review. We highlighted in green sections containing line-numbers, figure numbers and pages so that it will be easier to locate changes. We separated the response in two sections. The first one (current document) addresses the issues raised in the first round of comments, and the second addresses the issues regarding the second round, mainly referring to language issues and minor changes.

We sincerely hope this revised version reflects the transparency and quality work you are looking for.

**Section 1.**

**Rebuttal letter to the first set of comments.**

**The revised Tracked changes version contains colored in Blue the new additions, and in yellow the parts which we took from the final version. Furthermore, in this revised rebuttal letter we highlighted in green the additions which in essence are the association from each suggestion to the line number in which the change was performed. Unless specifically stated, this set of comments and replies is associated with the First and Original tracked changes document which is labeled as______________**

**Editorial Comments.**

The manuscript “Relationship between landslide susceptibility and social lag in Mexico City: The Case of the West Periphery” presents an interesting topic that integrates landslide susceptibility analysis with socio-economic factors. However, based on the two detailed reviews, there are significant methodological concerns that must be addressed before the manuscript can be reconsidered.

Key Issues to Address in the Revision:

**1. Justification of the Frequency Ratio (FR) Method:
o Reviewer 1 suggests that the use of FR needs stronger justification, especially given the availability of more advanced machine learning techniques (e.g., Random Forest, SVM, CNN).
o A comparative analysis should be conducted between FR and an ML-based model to demonstrate its effectiveness.**

Dear Editor and Reviewers, you are absolutely right, the justification presented to use the Frequency Ratio method is not enough. Consequently, we have now estimated 13 models to generate the susceptibility map, including but not limited to the ones suggested by the reviewers and editors e.g. Random Forest, SVM, Deep Learning and more. We present the models in page 8 beginning at line 312 all the way through line 392. This initial presentation refers to a general model introduction. Because of lack of space, Latex Editor moved a summary table below the section which includes each model‘s most important characteristics located in page 10, and since it is a Latex element it only used one numbered line, after line 418. Furthermore, model results which are the susceptibility maps are located in page 22 Figure 5 after line 802. In addition, the Champion model is depicted alone in Page 22 Figure 6 after line 809.

For each model we included the ROC curve and the AUC Value. A table containing these results is located in page 22 after line 797 and the Receiver Operating Curve (ROC) Plots are located in page 21 after line 791. This process allowed us to determine that Frequency Ratio was not the best model although it did a good job. We then changed Frequency Ratio by an XG Boost, which had the best performance according to the aforementioned metrics. The champion model was an XGBoost which is now used to generate the susceptibility map, as just mentioned, it is located on page 22 Figure 6 line 809.

**2. Weak Statistical Model for Social Lag Analysis:
o The multinomial logistic regression model shows an extremely weak fit (pseudo R² = 0.007352), making the relationship between social lag and landslide susceptibility statistically weak.
o Alternative modeling techniques (e.g., feature selection, interaction terms, or different regression models) should be considered.**

Dear Editor, thank you so much for this comment. You are so right, we have now moved entirely to a new and different approach, using SHAP values derived from a Random Forest model to visually determine the association magnitude and direction. We briefly describe SHAP values and the beeswarm plots in page 13 lines 486 - 498. In addition, SHAP plots are located in three sections: Full Dataset SHAP plots which, along with the corresponding explanatory text, are located in lines 957-986; CHangepoints section in lines 987-1038; and Decile section in lines 1040-1070.

Additionally, we used this model (A Random Forest) to calculate the R-squared metric. We anticipate that we got 80% R-squared, implying that, our analysis explained variance which is a decent statistic and which then provides credibility to our SHAP values. For Full dataset located in lines 958-962; Changepoints 992-997; and Decile separation in lines 1040-1048

Furthermore, for each partition we made we estimated Copula Models to provide a robust magnitude and direction metric for the association between Landslide Susceptibility and Social Lag.

Copula Theory can be found in lines 499-537. Estimations are separated in three sections which are Full dataset, changepoint induced partition and deciles. For the Full dataset, changes are in lines 974-986; for changepoint induced partitions changes are located in lines 1020-1038 ; and for decile partitions changes are in lines 1062-1070

**3. Validation of the Landslide Susceptibility Model:**
o **The lack of independent validation data is a major concern. It is crucial to include:
 A validation dataset of past landslides to assess accuracy.
 Performance metrics such as AUC-ROC curves to validate the predictive strength of the model.**

You are absolutely right. In this new version, we include validation metrics for each susceptibility map created by 13 models. We include ROC and AUC and throughout them, it was possible to discriminate models, and to finally select the XGBoost. These changes are incorporated in lines 393-408, which corresponds to the meaning of these variables, and the numerical/graphical implementations can be found in lines 787-802, there can be found a summary table containing AUC values as well as the actuall Matrix ROC plot for every implemented model. Furthermore, with respect to the independent validation for which a validation dataset is to be added, we included into the inputs repository the original World Bank Median from 1980-2018 about rainfall triggered landslide map (Median). The complete repository is open, free and fully accessible in Zenodo: https://doi.org/10.5281/zenodo.17156313

**4. Contradictions in Results and Hypothesis:**
**o The manuscript hypothesizes that higher social lag should correlate with higher landslide susceptibility, but findings show the opposite trend.
o A clearer explanation of this contradiction is required. If the hypothesis is incorrect, it should be revised with alternative theoretical support.**

Thank you very much for the kind comment. This new version changed completely the approach since the new models (Copula and SHAP plots derived from Random Forest) now have agreeable goodness of fit values (GoF) of R-squared around 0.8, and using these models with decent GoF there is no contradiction. This revised version not only includes new models to create susceptibility maps, it also includes new ways to assess the relationship we are studying: Landslide Susceptibility vs Social Lag, in this new version we found significant relations between the study variables provided that we partition the dataset by deciles and using a Changepoint algorithm. Both partitions allowed us to study this relationship with more granularity, and it helped to avoid noise that comes from studying a large heterogeneous area such as the western periphery of Mexico City. With this new approach, it was possible to find a positive association in many deciles and segments partitioned by the Changepoint algorithm. New association results can be found in lines 918-1070, including the following variants:

- Full dataset: lines 995-986
- Changepoint induced partitions: lines 987-1038
- Deciles: lines 1039-1070
- Individual association between landslide susceptibility and each of the variables with which the social lag index was built in line 1173.

**5. Choice of Huber Regression and Lack of Diagnostics:**
**o The rationale for using Huber regression should be justified over other robust regression techniques (e.g., Quantile Regression, Ridge Regression).
o The study lacks model diagnostics (e.g., residual analysis, VIF, or multicollinearity tests) to verify the robustness of Huber regression.**

Again, you are absolutely right. In this new version we changed the models and we included performance metrics. As stated before, we first used random forest to derive SHAP Values as an exploratory way to visually study the magnitude and direction of the relationship, for this model we included R-squared metric for each of the segements. Secondly, we used Copulas as a robust association method and include the Loglikelihood coefficient. As with the previous answer, we totally agree with the reviewers, which is why we totally dumped the Huber / Robust regression approach to the aforementioned SHAP and Copula approaches. New association results can be found in lines 918-1070, including the following variants:

- Full dataset: lines 995-986
- Changepoint induced partitions: lines 987-1038
- Deciles: lines 1039-1070
- Individual association between landslide susceptibility and each of the variables with which the social lag index was built in line 1173.

**6. Missing Data Issues and Temporal Bias in Data Sources:**
**o There is no explanation of how missing values in social lag variables were handled.
o The temporal mismatch between datasets (e.g., 2007 DEM vs. 2020 social data) could introduce bias.**

Thank you very much for this suggestion. You are absolutely right, in this new version the whole Data Sources section was re-written and we included every relevant detail that is required, including the missing values, an explanation about the temporal mismatch -we anticipate that the dataset was not so mismatched, the maps used were 2017 not 2007 as we initially claimed, it was an error on our side and we apologize. Additionally, we include data about the re-projection and methods used, we are sure this new section is totally illustrative about data’s metadata. To begin with, detailed data presentation begins at line 632 all the way through line 755. Specifically, an initial data presentation is located in lines 632-678. A Tau DEM presentation is in lines 679-755. We have now added a summary table with the input name, resolution, transformation and data source.line 751. Particularly, missing data is treated in lines 643, 670-676. Furthermore, the mismatch, which as we stated above, was a confusion on our side, is clarified in lines 640-641, and lines 649-654.

**7. Comparison with Official Landslide Hazard Maps:
o Given that Mexico City already has existing landslide hazard maps, the manuscript should compare its susceptibility map with official hazard maps to assess its reliability.**

Thank you very much. This is obviously a valid concern. We found the official CENAPRED’s landslide susceptibility map. It is a categorized version which means it colors risk-zones according to a pre-defined range. Since this is the official version, we compared our susceptibility map with this one to provide a better assessment. The resulting comparison between our generated categorized susceptibility map begins with the categorized susceptibility map in line 826 and the next paragraph which explains the similarity between lines 826-837. Furthermore, and more importantly, CENAPRED official susceptibility map is in line 837 Figure 9.

**8. Lack of Uncertainty Quantification:
o Landslide susceptibility models are probabilistic in nature. The study should provide confidence intervals for its estimates**.

We absolutely agree with this regard. To this end, we did the following “As a way to quantify uncertainty in the susceptibility map, we achieved this through a bootstrap resampling framework, which generates an ensemble of predictions to capture variability arising from sampling and model training processes. Specifically, we did 50 bootstrap iterations, wherein each iteration involves resampling the subsampled dataset with replacement to form a bootstrapped training set, followed by fitting an XGBoost classifier and predicting susceptibility probabilities across the cleaned feature matrix. The collection of these predictions, enables the derivation of ensemble statistics: the maximum (susceptibility_max) and minimum (susceptibility_min) values which represent the upper and lower bounds of prediction variability. These Max and Min maps make it straightforward to show the whole range of variability within the output.” This information is located between lines 810-819 with the associated plot which is in line 820 (Figure 7 Maximum and Minimum plots)

**9. Inadequate Literature Citations and Reference Formatting Issues:**
o **Reviewer 2 highlights improper references, missing citations from high-impact peer-reviewed sources, and unstructured reference formatting.
o The literature review should incorporate recent and relevant citations, including key works on landslide susceptibility modeling.**

Thank you very much for this comment. We revised our references and modified the main text and reference list accordingly; we added new ones and replaced the conflicting ones. The final version includes all of these changes. References are located in lines 1532 onwards. Noteice that Latex Editor overlapped two tables inside the references, but this will be edited and fixed in the final version.

**10. Data Resolution and Spatial Analysis Issues:**
**• There is no explanation of how datasets with different spatial resolutions (e.g., Landsat, MODIS, and DEM data) were standardized.
• Image enhancement or fusion techniques may be required to ensure data consistency.**

You are absolutely right. We re-write the vast majority of the Data Sources section to clarify exactly what we did about the spatial resolution, how it all fell into the same, comparable standard, the methods used, and the final resolution. We summarized all this information into a table at the end of this Data Sources section. An expanded explanation lies between lines 633-755. Specifically, in lines 636 we specify the reference coordinate system; line 649 specifies which images were not altered given they were at the same resolution. Lines 647-652 shows the transformations applied to this input; Lines 657-662 shows the transformation uses for WB data, as well as in lines 673-676. A summary is provided in lines 737-745 and Table 4 provides a summary which includes Resolution, transformation and source. This table is licated after line 751

**11. Missing Discussion:**
• **Sensitivity analysis should be performed to quantify the impact of different variables on landslide susceptibility.
• The manuscript lacks a proper discussion of computational efficiency, feature importance, and the limitations of the proposed method.**

Thank you for this comment. In the new version we added three dimensions of sensitivity analysis: for the susceptibility map model we added XGBoost Feature Importance. Also, we added the Information Gain Ratio, and the GeoDetector q-statistic. For the association between SLI and Landslide susceptibility, in one setting, we calculated dependence between each of the variables with which SLI was made and Landslide Susceptibility. The results are in plot after line 857, and the plot´s interpretation are between lines 838-857. As just stated, the techniques used are Information Gain Ratio, Feature Importance, and the q-statistic

Additionally, we included the limitations and efficiency in the XGBoost section since this was the model we used: “XGBoost complexity depends on the number of trees, features, tree-depth and data. It's parallel logic translates even complicated calculations into relatively simple threads. Although efficient, its limitations lie in that it has many hyperparameters to estimate, making it resource-intensive; it also tends to overfit data even with regularization; finally, it tends to be classified as black-box since the modeler is not fully aware of the model's inner calculations~\cite{bib72}”. This is located in lines 323-328

**12. Both reviewers express concerns about language clarity, grammatical errors, and ambiguous phrasing. A thorough English language revision is necessary to ensure the manuscript is clear, coherent, and professionally structured.**

I know, and I apologize about that! This version was reviewed several times, exclusively looking for grammar mistakes. We also tried to make sentences more concise. As of this last revision, we reviewed language again, we requested help from colleagues whom studied their graduate education in English language institutions. The last version presented before you is the result of this effort.

###################################################################

Additional Editor Comments:

The manuscript “Relationship between landslide susceptibility and social lag in Mexico City: The Case of the West Periphery” presents an interesting topic that integrates landslide susceptibility analysis with socio-economic factors. However, based on the two detailed reviews, there are significant methodological concerns that must be addressed before the manuscript can be reconsidered.

Key Issues to Address in the Revision:

**1. Justification of the Frequency Ratio (FR) Method:
o Reviewer 1 suggests that the use of FR needs stronger justification, especially given the availability of more advanced machine learning techniques (e.g., Random Forest, SVM, CNN).
o A comparative analysis should be conducted between FR and an ML-based model to demonstrate its effectiveness.**

Sir, thank you so very much for your comment. We agree that the use of FR method needs stronger justification. Yet, we did something bigger: as suggested, we calculated several other models-12 in total- to generate the susceptibility map, including many Machine Learning models, and we compared them using the ROC/AUC plot and metric. The champion model was an XGBoost, and the justification for using it now is that it has the highest AUC. Absolutely agree with you and thank you for pointing out to this weakness. The introduction to the new set of models we used lies within lines 312-392 as well as with a summary table in lines 418-419. Model results lie within lines 791-809, including Figure 5 in lines 802-803 which is a matrix plot containing every susceptibility map generated by each model.

A presentation to the performance metrics lies within lines 393-408. Actual performance metrics lie within lines 780-802, including Table 5 and Figure 4 in lines 797-798 and 790-791, respectively.

**2. Weak Statistical Model for Social Lag Analysis:
o The multinomial logistic regression model shows an extremely weak fit (pseudo R² = 0.007352), making the relationship between social lag and landslide susceptibility statistically weak.
o Alternative modeling techniques (e.g., feature selection, interaction terms, or different regression models) should be considered.**

This is an absolutely valid concern. To completely solve it we recalculated everything by using new association methods which are SHAP values derived from a random forest, and COPULAS, which are excellent at modeling non-linear associations. Furthermore, to delve deeper into our dataset, we partitioned it into deciles and also by using a Changepoint partitioning algorithm. For each of the resulting segments, we applied the SHAP and COPULA Analysis resulting in many significant relationships. Additionally, to assess how valid was the application of the method, we used the r-squared derived from the random forest as a fitting metric. En each of the segments, that metric rounded 80%, which is a decent fit. The whole association method lie within lines 918-1244

We estimated Random Forest models to eahc segment to then get the SHAP plots, each of the Random Forest model has a Rsqaured value close to 0.8

- Full dataset: line 959
- Changepoints: R-squared shown in Table 8 for each segment, lines 997-998
- Deciles: R-squared shown in Table 10 for each segment, lines 1048-1049

Specifically, we did several variants to make an analysis with different levels of granularity as follows:

Copula Results.

- Full dataset: using the complete dataset and calculating association via SHAP and Copulas lines 974-986 including Table 7 in line 987
- Changepoints: lines 1020-2038 including Table 9 in lines 1038.
- Deciles: Lines 1062-1071, including Table 11 in line 1070-1071

SHAP Results.

With respect to copula results they are located as follows:

- Full dataset: using the complete dataset and calculating association via SHAP is shown in lines 958-973 including Figure 13 in line 973
- Changepoint can be located within lines 998-1019 including Fig 14 in lines 998-1019.
- Deciles: 1049-1061, including Fig 15 in line 1061.

**3. Validation of the Landslide Susceptibility Model:
o The lack of independent validation data is a major concern. It is crucial to include:
 A validation dataset of past landslides to assess accuracy.
 Performance metrics such as AUC-ROC curves to validate the predictive strength of the model.**

Absolutely true, thank you for the comment. In this new version we included ROC-AUC plots and metrics for each of the models we used to create the landslide susceptibility map. These changes are incorporated in lines 393-408, which corresponds to the meaning of these variables, and the numerical/graphical implementations can be found in lines 787-802, there can be found a summary table containing AUC values as well as the actuall Matrix ROC plot for every implemented model. Furthermore, with respect to the independent validation for which a validation dataset is to be added, we included into the inputs repository the original World Bank Median from 1980-2018 about rainfall triggered landslide map (Median). The complete repository is open, free and fully accessible in Zenodo: https://doi.org/10.5281/zenodo.17156313

**4. Contradictions in Results and Hypothesis:
o The manuscript hypothesizes that higher social lag should correlate with higher landslide susceptibility, but findings show the opposite trend.
o A clearer explanation of this contradiction is required. If the hypothesis is incorrect, it should be revised with alternative theoretical support.**

Thank you so much again, and thanks for the patience of delving deeper into this point of our work. Yes, absolutely right, nevertheless we re-calculated the whole relationship with different methods and partitioning the dataset into deciles, with changepoints -and also doing it with the whole dataset. This new modeling approach did result into meaningful relationships which does not contradict the hypothesis. Thank you very much for the comment, and this limitation has been superseded.

Thank you very much for the kind comment. This new version changed completely the approach since the new models (Copula and SHAP plots derived from Random Forest) now have agreeable goodness of fit values (GoF) of R-squared around 0.8, and using these models with decent GoF there is no contradiction. This revised version not only includes new models to create susceptibility maps, it also includes new ways to assess the relationship we are studying: Landslide Susceptibility vs Social Lag, in this new version we found significant relations between the study variables provided that we partition the dataset by deciles and using a Changepoint algorithm. Both partitions allowed us to study this relationship with more granularity, and it helped to avoid noise that comes from studying a large heterogeneous area such as the western periphery of Mexico City. With this new approach, it was possible to find a positive association in many deciles and segments partitioned by the Changepoint algorithm. New association results can be found in lines 918-1070, including the following variants:

- Full dataset: lines 995-986
- Changepoint induced partitions: lines 987-1038
- Deciles: lines 1039-1070
- Individual association between landslide susceptibility and each of the variables with which the social lag index was built in line 1173.

**5. Choice of Huber Regression and Lack of Diagnostics:
o The rationale for using Huber regression should be justified over other robust regression techniques (e.g., Quantile Regression, Ridge Regression).
o The study lacks model diagnostics (e.g., residual analysis, VIF, or multicollinearity tests) to verify the robustness of Huber regression.**

Thank you for your valuable feedback. We agree that our initial use of Huber regression was a limitation, particularly regarding model diagnostics. To address this, we have replaced the Huber regression with a revised approach using SHAP values and Copula models. This change was made to enhance model interpretability and provide a more robust analysis. SHAP values directly address the lack of diagnostics by explaining the contribution of each feature to the model's output.

Regarding multicollinearity, we have maintained the exact covariates used by CONEVAL to ensure our findings are directly reproducible and comparable with the official Mexican Social Lag Index, which is a key objective of our study. This decision justifies including potentially collinear variables.

As with the previous answer, we totally agree with the reviewers, which is why we totally dumped the Huber / Robust regression approach to the aforementioned SHAP and Copula approaches. New association results can be found in lines 918-1070, including the following variants:

- Full dataset: lines 995-986
- Changepoint induced partitions: lines 987-1038
- Deciles: lines 1039-1070
- Individual association between landslide susceptibility and each of the variables with which the social lag index was built in line 1173.

**6. Missing Data Issues and Temporal Bias in Data Sources**:
**o There is no explanation of how missing values in social lag variables were handled.
o The temporal mismatch between datasets (e.g., 2007 DEM vs. 2020 social data) could introduce bias.**

Thank you. Actually, it was a mistake on our side since the dataset dates 2017 except for the Land Cover and CONEVAL social lag, which dates 2020. Nevertheless, we address this issue in the reformulated version of the Data Sources section. Furthermore, we made explicit what are the missing data code and how they were managed in the Data Sources section:

“Invalid no-data values, are excluded using a common mask, and any remaining missing values in predictor variables are imputed with their mean across valid observations, though this approach assumes random missingness and may overlook spatial correlations. As stated, we used subsampling to reduce computational demands by limiting the dataset to a predefined maximum number of pixels, using stratified sampling to maintain class balance between landslide and non-landslide instances, thus mitigating potential bias without introducing new missing data.”

As with the previous answer, we totally agree with the reviewers, which is why we totally dumped the Huber / Robust regression approach to the aforementioned SHAP and Copula approaches. New association results can be found in lines 918-1070, including the following variants: Full dataset: lines 995-986 Changepoint induced partitions: lines 987-1038 Deciles: lines 1039-1070 Individual association between landslide susceptibility and each of the variables with which the social lag index was built in line 1173.

Detailed data presentation begins at line 632 all the way through line 755. Specifically, an initial data presentation is located in lines 632-678. A Tau DEM presentation is in lines 679-755. We have now added a summary table with the input name, resolution, transformation and data source.line 751. Particularly, missing data is treated in lines 643, 670-676. Furthermore, the mismatch, which as we stated above, was a confusion on our side, is clarified in lines 640-641, and lines 649-654.

**7. Comparison with Official Landslide Hazard Maps:
o Given that Mexico City already has existing landslide hazard maps, the manuscript should compare its susceptibility map with official hazard maps to assess its reliability.**

Totally agree. This revised version includes a comparison with CENAPRED’s official map. CENAPRED is Mexico´s official disaster prevention agency, and we make the comparison with its categorical map, which is the only one they have publicly available. The resulting comparison between our generated categorized susceptibility map begins with the categorized susceptibility map in line 826 and the next paragraph which explains the similarity between lines 826-837. Furthermore, and more importantly, CENAPRED official susceptibility map is in line 837 Figure 9.

**8. Lack of Uncertainty Quantification:
o Landslide susceptibility models are probabilistic in nature. The study should provide confidence intervals for its estimates.**

Thank you very much for this comment. We totally agree with you and we now include an UQ framework as follows: “We absolutely agree with this regard. To this end, we did the following “As a way to quantify uncertainty in the susceptibility map, we achieved this through a bootstrap resampling framework, which generates an ensemble of predictions to capture variability arising from sampling and model training processes. Specifically, we did 50 bootstrap iterations, wherein each iteration involves resampling the subsampled dataset with replacement to form a bootstrapped training set, followed by fitting an XGBoost classifier and predicting susceptibility probabilities across the cleaned feature matrix. The collection of these predictions, enables the derivation of ensemble statistics: the maximum (susceptibility_max) and minimum (susceptibility_min) values which represent the upper and lower bounds of prediction variability.” This information is located between lines 810-819 with the associated plot which is in line 820 (Figure 7 Maximum and Minimum plots)

**9. Inadequate Literature Citations and Reference Formatting Issues:**
**o Reviewer 2 highlights improper references, missing citations from high-impact peer-reviewed sources, and unstructured reference formatting.
o The literature review should incorporate recent and relevant citations, including key works on landslide susceptibility modeling.**

Thank you very much for bringing this issue. The new version includes new references, and we also replaced the conflicting older references, thank you. The revised reference list begins at line 1532 onwards.

10. **Data Resolution and Spatial Analysis Issues:
• There is no explanation of how datasets with different spatial resolutions (e.g., Landsat, MODIS, and DEM data) were standardized.
• Image enhancement or fusion techniques may be required to ensure data consistency.**

We almost completely re-writte this whole Data Sources section to completely address this concern. The new version includes a very detailed description of the resolution, resampling and an explanation about the data resolution apparent mismatch, we addressed every concern raised within this section. An expanded explanation lies between lines 633-755. Specifically, in lines 636 we specify the reference coordinate system; line 649 specifies which images were not altered given they were at the same resolution. Lines 647-652 shows the transformations applied to this input; Lines 657-662 shows the transformation uses for WB data, as well as in lines 673-676. A summary is provided in lines 737-745 and Table 4 provides a summary which includes Resolution, transformation and source. This table is licated after line 751

11**. Missing Discussion:**
**• Sensitivity analysis should be performed to quantify the impact of different variables on landslide susceptibility.
• The manuscript lacks a proper discussion of computational efficiency, feature importance, and the limitations of the proposed method.**

We are deeply thankful about bringing this concern. As stated before, in the new version we added three dimensions of sensitivity analysis: for the susceptibility map model we added XGBoost Feature Importance. Also, we added the Information Gain Ratio, and the GeoDetector q-statistic. For the association between SLI and Landslide susceptibility, in one setting, we calculated dependence between each of the variables with which SLI was made and Landslide Susceptibility.The results are in plot after line 857, and the plot´s interpretation are between lines 838-857. As just stated, the techniques used are Information Gain Ratio, Feature Importance, and the q-statistic

Additionally, we included the limitations and efficiency in the XGBoost section since this was the model we used: “XGBoost complexity depends on the number of trees, features, tree-depth and data. It's parallel logic translates even complicated calculations into relatively simple threads. Although efficient, its limitations lie in that it has many hyperparameters to estimate, making it resource-intensive; it also tends to overfit data even with regularization; finally, it tends to be classified as black-box since the modeler is not fully aware of the model's inner calculations~\cite{bib72}”. This is located in lines 323-328

**12. Both reviewers express concerns about language clarity, grammatical errors, and ambiguous phrasing. A thorough English language revision is necessary to ensure the manuscript is clear, coherent, and professionally structured.**

Thank you very much for this comment. We proofread the final version several times to address this issue and to make this final version as best as possible in terms of language formality. The last version was totally revised and corrected, thank you so very much for raising this issue.

[Note: HTML markup is below. Please do not edit.]

Reviewers' comments:

Reviewer's Responses to Questions

**Comments to the Author**

1. Is the manuscript technically sound, and do the data support the conclusions?

The manuscript must describe a technically sound piece of scientific research with data that supports the conclusions. Experiments must have been conducted rigorously, with appropriate controls, replication, and sample sizes. The conclusions must be drawn appropriately based on the data presented.

Reviewer #1: Partly

Reviewer #2: No

2. Has the statistical analysis been performed appropriately and rigorously?

Reviewer #1: Yes

Reviewer #2: No

3. Have the authors made all data underlying the findings in their manuscript fully available?

The [PLOS Data policy](http://track.editorialmanager.com/CL0/http:%2F%2Fwww.plosone.org%2Fstatic%2Fpolicies.action%23sharing/1/010f019587b87f70-c58336c5-a7bf-4874-9306-46227e717f6d-000000/35_96JRYjzWL0enChmsUYvGKCyL6bioo3QygSYnSNjM=201) requires authors to make all data underlying the findings described in their manuscript fully available without restriction, with rare exception (please refer to the Data Availability Statement in the manuscript PDF file). The data should be provided as part of the manuscript or its supporting information, or deposited to a public repository. For example, in addition to summary statistics, the data points behind means, medians and variance measures should be available. If there are restrictions on publicly sharing data—e.g. participant privacy or use of data from a third party—those must be specified.

Reviewer #1: Yes

Reviewer #2: No

Kindest sirs, I just want to emphasize that our whole dataset is available in a ZENODO repository, which is always free open access. That is, anyone can see and use the inputs we used. Originally, we had it as a link to Google drive, but for the last version we, moved it to ZENODO, the link to the inputs is here and the link also appears in the final pdf we are handing over:

https://zenodo.org/records/17156313

With DOI identifier: 10.5281/zenodo.17156312

4. Is the manuscript presented in an intelligible fashion and written in standard English?

PLOS ONE does not copyedit accepted manuscripts, so the language in submitted articles must be clear, correct, and unambiguous. Any typographical or grammatical errors should be corrected at revision, so please note any specific errors here.

Reviewer #1: No

Reviewer #2: No

**Reviewer #2: A super simple straightforward application of FR for landslide hazard in a disorganized and unstructured draft.

English proficiency stands beyond quality due to frequent flaws, vague statements, linguistic flaws…**

Dear reviewer, your kind comment was taken into account, and the whole document was revised. Thank you for your comment. We have addressed this issue by proofreading and correcting every grammatical error found in the text. Hope the new version is sufficiently improved in terms of language.

Furthermore, as you correctly noticed, the first version included a simple Frequency Ratio implementation, yet this new version was heavily upgraded and now includes 13 models among which lie some of the most recent Machine Learning implementations such as XGBoost, Random Forest, Support Vector Machine, and more. Furthermore, it now includes performance metrics and plots such as ROC plots, as well as a matrix containing the susceptibility map generated by each of the algorithms. This addition lies in lines 787-809. Particularly,

- There is a first model presentation within lines 312-392.
- Model metric’s presentation lies within lines 394-408.
- Summary model table with each model most important characteristics is in page 11.
- Model results begin in line 787 with each model’s ROC and AUC in Figure 4 line 790.
- Table 5 in line 787-798 presents AUC results
- Figure 5 in lines 802-803 contains results for each of the models used
- Line 809 Figure 6 contains the susceptibility map generated by the champion model, an XGBoost.

**Improper references and commercial weblinks while solid publications are missing. Using a dissertation for FR from 2018 while this method has many solid references, ….**

Thank you very much for this comment. You are absolutely right. We have added new references, we have changed the conflicting old references to make the new version solid., Again, thank you so very much for noting this issue, the upgraded references does not include this type of references anymore. New references begin in line 1532

**The next critical issue is the perquisite use of unified pixel size, and therefore it cannot be functional for extracted attributes from different imageries like Landsat, Modis, and … with different resolutions incorporated with raster data.

Therefore, based on the used datasets, it can only be acceptable when the inputs don’t change, else in the case of modified images (in pixel or grids) it will no longer match and hence, the integrity cannot be verified. In addition, the overlaying the raster and vector with different resolutions and geospatially distribution requires image enhance processing, or image fusion Technique. Novel approach can be found at** [**https://www.sciencedirect.com/science/article/abs/pii/S0341816219303674**](https://www.sciencedirect.com/science/article/abs/pii/S0341816219303674)**,** [**https://doi.org/10.1155/2023/9429505**](https://doi.org/10.1155/2023/9429505)**, …**

Thank you very much for this issue. We have rewritten the entire Data Sources section to make explicit every detail with respect to the data sources, including resampling methods, projection, final resolution, etc. Sir, your concern is of utmost importance for us. In the new Data Sources section, within lines 632-749 we address the issues you just raised. Particularly, we state that some of the inputs were already in the same resolution (30m), and those that were altered were Land Cover, which was reprojected and resampled. We used bicubic interpolation with anti-aliasing (lines 647-652). Landslide inventory and the mask that was built with it, was rasterized, resampled, and reprojected using nearest-neighbor resampling (lines 656-659). Further subsampling was used and is presented in lines 673-676. An additional summary paragraph is presented at lines 737-744, and also, we created a table which concentrates Resolution, transformation and source, this table begins in line 751.

**Without any doubt, REJECTED conclusion. 1) pretty long and unjustified, 2) it obviously is not the place for citation**

Thank you very much for your comment. The new version has a revised shorter conclusion wich only addresses the central points in a straightforward way. Again, thank you so much for your comment. We agree with the issue you just raised and the new version shortened the conclusion, and we removed any citations right since the beginning. Conclusion can be read in lines 1437-1463.

**Overall,
1. Lack of novelty is concrete and beyond taking time for discussion**

Thank you very much for your point of view. There are not too many works that look for relationships between landslide susceptibility and social lag. In the new version we included new techniques to find an association between landslide susceptibility and social lag along dataset partitioning techniques. These techniques, which are Copula models and SHAP values allowed us to find meaningful relationships in important segments of the dataset. We do apologize that the first version lacked the novelty we bring about in the new version which as stated, includes the most innovative Machine Learning (ML) models making a total of 13 models considered (for which the plot is located in line 802) that produce the susceptibility maps as well as with the aforementioned Copula and SHAP plots which are excellent methods to detect non-linear relationships between variables. This section is located in lines 955-1070 for the full dataset, changepoint induced partition and by deciles. Furthermore, we used these same techniques to look for potential association for each of the variables conforming the social-lag index and landslide susceptibility which can be foun in lines 1184-1224. Furthermore, Few studies use the Tau DEM suite, which is ideal to better model earthflow landslides. Additionally, few studies, separate the study region into sections to analyze in a more granular way the stories that develop en each section. We did this by means of Changepoint algorithm partitioning and by decile subdivision. Together, the use of ML models, the use of non-linear Copula and SHAP plots, Changepoint and Decile partitioning, and the use of Tau DEM conform an approach rarely seen in literature, which is the novelty we bring about.

**2. Doesn’t provide proper analyzed research gaps, taxonomy, and meaningful guidelines in the domain.**

We acknowledge this concern and have revised the introduction and discussion to explicitly map existing studies into a clear taxonomy (physical-only, social-only, and integrated approaches). Within this framework, we identify the research gap our work addresses: the lack of reproducible, explainable models that jointly assess landslide susceptibility and social lag at the urban periphery. We also provide methodological guidelines, emphasizing data harmonization, explainable ML (SHAP), and robust validation. Thank you very much for bringing this topic up. We are sorry that the initial version was disappointing, yet in this new version we emphasize that, although there are studies addressing the technicalities of how to create better landslide susceptibility models, we emphasize the association between landslide susceptibility and social variables as our core study object, which is not widespreadly exploited in literature. We also emphasize that there are previous studies already using social variables, which we cite, and from which we collect the association direction e.g. higher susceptibility implies more social lag and viceversa. This part lies in lines 128-158. Furthermore, we created a table summarizing the literature findings (Table 1, line166).

**3. In comparison with advanced approaches For example,** <https://www.sciencedirect.com/science/article/abs/pii/S0341816219303674>, <https://link.springer.com/article/10.1007/s10346-019-01299-0>, <https://link.springer.com/article/10.1007/s100640050066>, <https://www.tandfonline.com/doi/abs/10.1080/02723646.2021.1978372>, <https://www.sciencedirect.com/science/article/pii/S2590056022000202>, <https://www.tandfonline.com/doi/abs/10.1080/01431161.2019.1672904>, … **it is uncompetitive.**

Thank you for pointing us to these advanced methods. We agree they represent strong state-of-the-art lines of work. Our goal in this paper is, rather than proposing a new algorithm, we provide an interpretable, policy-oriented framework that links landslide susceptibility to social lag using some of the most advanced Machine Learning methods to create the susceptibility map as well as innovative method to associate results with the social realm, these are Copulas and SHAP plots:

1. Copula-based dependence analysis,
2. SHAP explanations, and
3. Changepoint/decile partitioning to expose spatially heterogeneous regimes.

Overall association between landslide susceptibility and social lag index in its variants lies between lines 918-1070. Furthermore, the association between landslide susceptibility and each of the variables conforming the social-lag index lies within lines 1118-1223.

This combination is, to our knowledge, uncommon in the Mexico City context and is designed to be reproducible with widely available data and transparent for risk-management decisions. To address your concern and improve comparability, the revision includes:

- Stronger baselines and fair evaluation for which we created an AUC plot for each model and is presented in Figure 4 lines 790-791 and Table 5 lines 797-798.
- We report XGBoost/LightGBM/Random Forest with spatial block cross-validation, AUC and ROC. Table 5 starting in lines 797 present the AUC values by model and the ROC plots for each of the model are available in Figure 4 in line 790

What our approach adds beyond accuracy. We truly do not mean to be repetitive nor annoying, but to comply with the mandate of signaling where each part of the rebuttal letter corresponds to the tracked change document, we clarify that the empirical association methods begin at line 918 with the association using copulas and SHAP values for the full dataset, changepoints induced partitions and decile partitions. Furthermore, these same techniques are applied to study the association between susceptibility and each of the variables with chi the social lag index was built. This approach starts in line 1167 all the way through line 1223 including Table 14.

- Copulas: quantification of dependence strength and direction by segments/deciles (including tail-sensitive diagnostics). We partitioned results in Full dataset, Changepoint partitions, and Decile partitions located in lines for the Full dataset in lines 974-986 including Table 7 in line 986 which summarize copula results; For Changepoint partition results lie in lines 1020-1038 including Table 9 in line 1038 summarizing copula results; for Decile partition it begins in line 1061 to 1070 including Table 11 with copula results for each decile.
- SHAP: local/global interpretability that consistently elevates educational deprivation and basic-infrastructure deficits, making the results auditable by stakeholders. We partitioned results in Full dataset, Changepoint partitions, and Decile partitions located, for the Full dataset in lines 962-973, including Figure 13 lines 973-974; for changepoint partition results lies in lines 998-1019 Including Figure 14 in line 1019; and for decile partitions in lines 1049-1061, including Figure 15 showing SHAP plots for every decile (lines 1061-1062).
- Partitioning (changepoints/deciles): reveals regimes where high SLI coincides with high susceptibility versus resilient, self-contained communities—insights that black-box models often do not make explicit. Mentioned and referenced to number lines just above.
- Furthermore, we included SHAP and copula results for eahc of the variables with chich the social lag index was built, as well as Feature importance. SHAP explanation lies within lines 1184–1205, including Figure 18 in line 1205-1206. Copula results lie un lines 1206-1223
- Transparency and reproducibility.

In sum, we position the paper not as an algorithmic innovation to the most advanced pipelines, but as a complementary, interpretable framework that connects physical susceptibility with social vulnerability and yields actionable diagnostics. We believe these additions address the competitiveness concern while preserving the paper’s primary contribution. Nevertheless, we included some of the most advanced ones with high reliability Machine Learning methods available, such as Extreme Gradient Boosted Trees, Support Vector Machine, and Deep Learning, among others. A brief model presentation lies in lines 312-392, susceptibility maps generated by each of the models are in plot 5 after line 802.

With all these new additions, which include 13 Models including Machine Learning models, innovative association methods such as SHAP and Copulas, and the partitioning methods to go granular and deep into the intricacies of our dataset we believe our work is now well positioned.

**4. Lack of data analysis/data visualization in considering the selected attributes are opaque**

Thank you again for your comment. We regret that the older version did not include this. Nevertheless, the new version makes this explicit, we provide explicit visualization of each of the inputs used -DEM, Slope, Aspect, etc- along with the way each of the input was chosen and pre-processed (e.g. resampled) to become a valuable input for our analysis. Again, we are sorry that this was not done from the beginning. Again, we are sorry for not having included these plots earlier. Figure 1 contains a matrix plot with every input used in the study, ranging from DEM, Aspect, Roughness to the newly added TAUDEM. These plots are located in lines 751-752.

**5. Lack of considering the effect of subsurface spatial soil/rock type distributions** (<https://link.springer.com/article/10.1007/s10064-018-1400-9>, <https://www.sciencedirect.com/science/article/abs/pii/S0169555X16306419>, <https://link.springer.com/article/10.1007/s10346-013-0409-1>, <https://www.sciencedirect.com/science/article/abs/pii/S0013795224002655>, <https://www.mdpi.com/2220-9964/10/5/341> ...), considering the clay sensitivity and its effect in landslide (<https://www.sciencedirect.com/science/article/pii/S0037073817303019>; <https://www.sciencedirect.com/science/article/abs/pii/S0013795215000411>, <https://www.sciencedirect.com/science/article/pii/S0341816222002752> …), spatial analysis of the collected data and involved uncertainty of the post processing geo-model (<https://link.springer.com/article/10.1007/s00366-023-01852-5>, …

Thank you for your kind comment, this approach would be great for another paper, to include subsurface distribution sounds paramount, but it was not the goal of our current research to include it. Furthermore, this input is not necessarily required in other papers already published by PlosONE, for instance:

<https://journals.plos.org/plosone/article?id=10.1371/journal.pone.0292897>

<https://journals.plos.org/plosone/article?id=10.1371/journal.pone.0245668>

Additionally, the new version includes a bootstrap resampling framework that generates ensemble predictions to model variability from sampling and training. These predictions made it possible to generate ensemble statistics: maximum /minimum susceptibility maps. In the new version we implemented a bootstrap resampling that generates ensemble predictions (50 bootstrap iterations) and each within each iteration there is a resampling of teh subsampled DB with replacement to get the training set. After that, an XGBoost model is estimated to predict susceptibility probabilities. The collection of these probabilities allows to get the maximum and minimum ensemble statistics. The text associated with this approach lies between lines 810-819, and the Maximum-Minimum plots lie in line 820-821 Figure 7 (Maximum and Minimum plots)

**6. You have different attributes. The sensitivity analysis and predictability SHOULD be carried out via weight database of the optimum model to show the importance of each selected features (Search for updating the neural network models using different sensitivity analysis methods, sensitivity analysis for neural networks, novel feature selection using sensitivity analysis…)**

Thank you very much!! You are absolutely right in this issue. The new version includes Feature Importance, Information gain, and q-statistic, which we believe will totally fill this gap. This begins in line 838 to 857 including Figure 10 (Feature Importance plots) on line 857

**7. Lack of any convincing and documented Discussion cannot be neglected. Solid comparison with other approaches and scholars/visualized results in compare with other scholars/computational time and cost based on the system and model used/technical limitations/uncertainty quantifications comparing with advanced automated predictive deep learning models..., impact of bias of the used data on the results, noise strategy removal, …**

Thank you very much for your concern. The new version includes a totally re-written discussion which addresses the central points of the results, providing explanations. We also compared our results with those of the official Mexican Agency for Disaster Prevention (CENAPRED), they generated a categorized susceptibility map which we included into our new version. Additionally we include the model (XGBoost) technical limitations, Uncertainty Quantification and Imputation methods, when those were necessary. Again, thank you very much for your input. The whole discussion was changed given your request and given that we fully modified the research by adding new Machine Learning models as well as new association techniques such as Copulas and SHAP values to interpret different partitions (Full dataset, Changepoint partition, and Decile partition). The New discussion section lies within lines 1261-1372.

Furthermore, we agree that a more explicit and structured Discussion is useful. We have substantially expanded the Discussion and limitations, and we now address each of the reviewer’s points as follows:

1. “Solid comparison with other approaches and scholars / visualized results in compare with other scholars”
    In the original version, the comparison with previous work was concentrated in the Literature background section and Table 1. We added a new paragraph where we contrast our main findings with key studies that integrate landslide susceptibility and social variables in the Himalayas, Brazil, Ecuador and Guerrero, Mexico, located in lines 141-158. Furthermore, in the Discussion section we now state that high physical hazard frequently overlaps high social disadvantage in peripheral or informal areas. We also highlight how our use of SHAP values, copulas and decile/changepoint segmentation moves beyond the overlay-based approaches used in these works, providing formally quantified associations.
2. Regarding “visualized comparison”, direct pixel-wise comparison with other scholars’ maps is not possible because each study uses a different region, scale and input data. Instead, we now emphasize the comparative evidence provided by our own Figure 5, which visually contrasts the susceptibility patterns produced by 13 Machine Learning models, including deep learning, FR, WOE and logistic regression, at the same resolution and using the same inputs. This figure, together with Table 5, illustrates how our selected model (XGBoost) behaves relative to other approaches used in the landslide literature.
3. “Computational time and cost based on the system and model used”
    We agree that it is useful to briefly document computational demands. In the revised Methods we now describe the hardware and software environment. Specifically, we report the approximate time needed to run everything to generate susceptibility predictions for the full grid on our workstation. These values show that the proposed workflow is computationally feasible on standard research hardware. Because previous landslide-susceptibility studies rarely report comparable timing information and use heterogeneous implementations and hardware, we restrict ourselves to reporting absolute times for our own runs and avoid cross-study timing comparisons, which would be misleading. This information is located in lines, **540-554 of the last tracked changes version, that is, this section is not on the original tracked changes, it is contained in the last version we just uploaded to PLOS One system.**
4. “Technical limitations / impact of bias of the used data on the results / noise strategy removal”
    We appreciate this important point. We have added a dedicated subsection at the end of the document titled “Limitations” in lines 1216-1229 of the nw tracked changes version. There we summarize several sources of potential bias and noise that were previously mentioned only in passing:
   - (i) The World Bank Global Landslide Catalog was originally produced at ~1 km resolution and is known to be spatially incomplete; after downscaling to 30 m, pixel-level landslide locations retain positional uncertainty despite our use of nearest-neighbour resampling.
   - (ii) The CONEVAL social lag information is available only at the AGEB level and, as we already note in the Introduction, may fail to capture highly disadvantaged pockets inside otherwise “low-lag” units, biasing the index downward in unequal areas.
   - (iii) DEM and land-cover layers correspond to 2017 and 2020 respectively, whereas landslides span 1980–2018, which introduces some temporal mismatch.
   - (iv) Missing predictor values are handled using a common mask and mean imputation; we now explicitly state that this assumes missing-at-random and may attenuate extreme values.
5. We also clarify which noise-mitigation strategies we already employed—consistent resampling of all layers to a common grid, removal of invalid NoData pixels, stratified sampling to limit class imbalance, and the use of robust tree-based models (lines 670-678)—and we stress that, despite these measures, residual bias due to inventory incompleteness and social-data aggregation remains and should be considered when interpreting the results.
6. “Uncertainty quantifications comparing with advanced automated predictive deep learning models…”
    In the Tracked Changes first revised manuscript we already provided an uncertainty analysis for our final susceptibility map based on a 50-run bootstrap ensemble of XGBoost models, yielding Max/Min maps that summarize prediction variability across the study area.
7. The reviewer additionally suggests comparing this UQ with that of “advanced automated predictive deep learning models”. Our experimental pipeline included a deep neural network classifier trained on exactly the same inputs as the other models, and its ROC-AUC performance is already reported in Table 5. However, implementing a full bootstrap-based UQ for all 13 models, including the deep network, would multiply the computational cost and substantially increase the length and complexity of the paper. We therefore chose to apply the bootstrap to the champion model (XGBoost), which slightly outperforms the deep network and other competitors according to ROC–AUC, and to interpret the resulting uncertainty maps as indicative of the variability of a high-performing model, lines 810-819 of the original tracked changes version.

Overall, we believe that these additions result in a clearer and more convincing Discussion, making explicit how our work relates to the existing literature, how computationally feasible the workflow is, and which data limitations and uncertainties remain.

**8. Trivially ill-formatted reference list**

Thank you very much for your concern. We have addressed this issue and the new work contains new reference and we have removed the old conflicting references. The new reference list begins in line 1532.

**Section 2.**

**Rebuttal letter to the second set of comments.**

**Dear Editor and Reviewers. The following set of comments refers to the second review. Mapping from this comments to numbered lines will be made into a second tracked changes document titled_______**

Acceptance however, should be subject to:

- Response-to-review table submission Full mapping of all comments made by each prior reviewer/editor to certain line numbers and text modification in the revision,
- One last pass of professional editing, preferably by a native or an identified editing agency,
- Fixing of small textual problems indicated by Reviewer 3 (e.g., typos, figure captions, method/parameter transparency, and suggestion of future work).

In case of these conditions, it may be possible to proceed with the acceptance, the technical basis is now satisfactory and the work has methodological significance to policy and disaster risk mitigation in the urban peripheral setting.


[Note: HTML markup is below. Please do not edit.]

Reviewers' comments:

Reviewer's Responses to Questions

Comments to the Author

1. If the authors have adequately addressed your comments raised in a previous round of review and you feel that this manuscript is now acceptable for publication, you may indicate that here to bypass the “Comments to the Author” section, enter your conflict of interest statement in the “Confidential to Editor” section, and submit your "Accept" recommendation.

Reviewer #2: (No Response)

Reviewer #3: All comments have been addressed

2. Is the manuscript technically sound, and do the data support the conclusions?

The manuscript must describe a technically sound piece of scientific research with data that supports the conclusions. Experiments must have been conducted rigorously, with appropriate controls, replication, and sample sizes. The conclusions must be drawn appropriately based on the data presented.

Reviewer #2: No

Reviewer #3: Yes

3. Has the statistical analysis been performed appropriately and rigorously?

Reviewer #2: No

Reviewer #3: Yes

4. Have the authors made all data underlying the findings in their manuscript fully available?

The [PLOS Data policy](http://track.editorialmanager.com/CL0/http:%2F%2Fwww.plosone.org%2Fstatic%2Fpolicies.action%23sharing/1/010f019a99e0ad17-3922fb5c-4a14-49d2-86b8-f8390b93a9ca-000000/h8lnvlJyuTocp5j1SOiSt5beIb8ZPKiUiHoHm1yqrLM=238) requires authors to make all data underlying the findings described in their manuscript fully available without restriction, with rare exception (please refer to the Data Availability Statement in the manuscript PDF file). The data should be provided as part of the manuscript or its supporting information, or deposited to a public repository. For example, in addition to summary statistics, the data points behind means, medians and variance measures should be available. If there are restrictions on publicly sharing data—e.g. participant privacy or use of data from a third party—those must be specified.

Reviewer #2: No

Reviewer #3: Yes

5. Is the manuscript presented in an intelligible fashion and written in standard English?

PLOS ONE does not copyedit accepted manuscripts, so the language in submitted articles must be clear, correct, and unambiguous. Any typographical or grammatical errors should be corrected at revision, so please note any specific errors here.

Reviewer #2: No

Reviewer #3: Yes

6. Review Comments to the Author

Please use the space provided to explain your answers to the questions above. You may also include additional comments for the author, including concerns about dual publication, research ethics, or publication ethics. (Please upload your review as an attachment if it exceeds 20,000 characters)

Reviewer #2: WONDERING. None of the commente neither are responded propoerly nor analytically discussed.
A simple evidence can be assigned to not revised Englsih as trivailly suffers from incohesion and inconssitent use of the third passive. Missing several comments, missing assigned line number corresponding to each comment or modification palce, pretty unjustified and long conclusion, …

Sir, thank you so very much for your kind comment. We added a full discussion to the set of comments as requested (**these comments are in Section 1 of this document**), we do hope you like them since we sincerely intend to give you the best possible response given our knowledge. Additionally, we mapped each of the comments to line numbers for better and rapid assessment. Furthermore, we agree with you that the conclusion was long, therefore, we extracted the most relevant features to summarize it into a relevant yet small piece of text. Finally, a full text revision was made by ourselves and by colleagues whom study their Ph.D´s in an English native nation, therefore, we hope this last version will fulfill your expectations. These last final changes are available on the **last Tracked Changes version we just uploaded to PLOS ONE system, not in the original tracked changes document**.

Reviewer #3: The authors have substantially revised the manuscript, addressing the major methodological concerns from the initial review. The integration of multiple machine learning models for susceptibility mapping, with XGBoost emerging as the top performer (AUC = 0.883), is a strong improvement. Validation through ROC/AUC curves, uncertainty quantification via bootstrap resampling, and comparison with the official CENAPRED map enhance the reliability of the results. The shift to SHAP values and Copula models for analyzing the relationship between landslide susceptibility and social lag provides a more nuanced, granular perspective, revealing regime-specific associations that were absent in the original version.
The literature review now includes recent, high-impact citations, and reference formatting has been corrected. Data handling details (e.g., resolutions, resampling, missing values) are clearly described. The discussion is more focused and interpretive, and the conclusion is concise.
The revisions resolve the key issues, making the manuscript suitable for publication with minor changes:

Correct the typo "socila lag" in the abstract to "social lag". Typo corrected, thank you so very much for noticing, Sir!! This change is not associated with a line number in the PLOS One LaTex template since it is the abstract, yet, the change was made as suggested.

In the introduction (lines 81-82), rephrase for clarity: "as global warming intensifies and climatic events become more extreme, such as precipitation" to "as global warming intensifies extreme precipitation events". Thank you so very much for your kind suggestion, sir, this change lies in lines 80-81 on the last tracked version document.

Ensure all figure captions are descriptive and consistently formatted (e.g., Fig. 3: "Correlation matrix among environmental inputs used for susceptibility modeling"). Thank you very much for your kind suggestion. In the last version, every Figure’s caption was revised and corrected, thank you so very much!!!

In the methods, briefly note key XGBoost hyperparameters (e.g., n_estimators=100, learning_rate=0.1) for reproducibility. Thank you very much for your kind suggestion, this information is now included between lines 328-334 of the last tracked changes document, which corresponds to the Materials and Methods section, thank you!

Add a sentence to the conclusion suggesting future work, such as validating the model with real-time landslide data or incorporating climate projections. We created a Future Work section at the end of the document in lines 1232-1240 containing this suggestion, thank you so very much!

I recommend acceptance after these minor revisions.

7. PLOS authors have the option to publish the peer review history of their article ([what does this mean?](https://track.editorialmanager.com/CL0/https:%2F%2Fjournals.plos.org%2Fplosone%2Fs%2Feditorial-and-peer-review-process%23loc-peer-review-history/1/010f019a99e0ad17-3922fb5c-4a14-49d2-86b8-f8390b93a9ca-000000/RGz_m4K06o59S0ciLoMmQ_77ZXOStJjKCNMoSUZHtgM=238)). If published, this will include your full peer review and any attached files.


If you choose “no”, your identity will remain anonymous but your review may still be made public.


Do you want your identity to be public for this peer review? For information about this choice, including consent withdrawal, please see our [Privacy Policy](https://track.editorialmanager.com/CL0/https:%2F%2Fwww.plos.org%2Fprivacy-policy/1/010f019a99e0ad17-3922fb5c-4a14-49d2-86b8-f8390b93a9ca-000000/50ACSCJoq77iqwOPP5rQSo4wNLoG-a6nP9nVH8acUKs=238).

Reviewer #2: No

Reviewer #3: No
