# Supplementary material for: Relationship between landslide susceptibility and social lag in Mexico City: The case of the west periphery
Source: PLoS One. 2026 Jan 9;21(1):e0340639. doi: 10.1371/journal.pone.0340639 (PMC12788685; doi:10.1371/journal.pone.0340639)
Supplement: Attachment — Submitted filename: Response_to_Reviewers_auresp_3.docx [file pone.0340639.s005.docx]

**Kindest Sirs,**

We are deeply thankful for your quick and kind reply. We have prepared a list with numbered lines with respect to each of the suggested changes. Again, we are deeply thankful for the efforts you’ve made to improve so much the quality of our work.

**Changes received on December 18 2025:**

**1. Can you please upload an additional copy of your revised manuscript that does not contain any tracked changes or highlighting as your main article file. This will be used in the production process if your manuscript is accepted. Please amend the file type for the file showing your changes to Revised Manuscript w/tracked changes. Please follow this link for more information:** [**http://blogs.PLOS.org/everyone/2011/05/10/how-to-submit-your-revised-manuscript/**](http://blogs.plos.org/everyone/2011/05/10/how-to-submit-your-revised-manuscript/)**. We noticed that some parts in your References section are boxed in color blue.**

Dear sirs, the new **Manuscript.pdf** without any tracked changes nor highlighting was uploaded to PLOS ONE system. This included the removal of the blue boxed in the References section. Thank you.

**2. Please upload a new copy of your Figures as the details is not clear. Please follow the link for more information:** [**https://journals.plos.org/plosone/s/figures**](https://journals.plos.org/plosone/s/figures)**. Kindly change your figure description in your file inventory for us to determine which is figure 1-14.**

Dear Sirs, every figure was assigned Tiff format and each figure name now corresponds to how it is referenced in text e.g. Fig 1, Fig 2 and so on.

**3. Please ensure that you refer to Tables 14 and 15 in your text as, if accepted, production will need this reference to link the reader to the Table.**

Thank you very much for this comment, we now cross-reference to Tables 14 and 15 in page 27 lines 953-954 in the manuscript.pdf document.

################### Changes suggested on December 15 2025 ###################

- **Although language has improved, some sentences remain long or awkward and would benefit from one more round of careful English editing for clarity and conciseness.**

Thank you so very much for your kind comment. The whole article was completely revised, and to resolve the issues associated with this comment, the following changes were implemented:

**Grammatical issues.**

Abstract: we corrected earthflow to earthflow.

Lines 303-304. “The methods here presented” / to / “The methods presented here”.

Lines 513-514. “they allow to model” / to / “they allow researchers to model”.

Lines 530-531. “Table 3 engulfs” / to / “Table 3 summarizes”.

Lines 538-539. “we did’t used GPU’s” / to / “we didn’t use GPUs”.

Line 698 . “every tested models” / to / “every model tested”

Lines 776-777. “within the each AGEB” / to / “within each AGEB”.

Line 791. “Susceptibilitytends” / to / “Susceptibility tends”.

Line 454. “Find -if there exist- a relationship” / to / “find whether there exists a relationship”.

Lines 624-625. “the D-Infinity algorithm, offers on one hand, an advanced approach ” / to / “the D-Infinity algorithm, offers an advanced approach”.

**Long paragraphs that were separated and shortened**

Lines 30-37. Paragraph made concise.

Lines 46-51. Paragraph split and shortened.

Lines 62-68. Paragraph shortened.

Lines 69-71. Paragraph shortened.

Lines 102-110. Paragraph split.

Lines 123-130. Paragraph split.

Lines 137-141. Paragraph shortened.

Lines 141-152. Paragraph split.

Lines 198-201. Paragraph modified for conciseness.

Lines 232-242. Paragraph split.

Lines 603-608. Paragraph shortened.

Lines 611-620. Paragraph shortened.

Lines 784-798. Paragraph changed for consistency.

Lines 855-863. Paragraph split.

Lines 1101-1115. Paragraph split.

Lines 1125-1137. Paragraph split.

- **Ensure that all tables and figures are properly placed and numbered in the final layout (some overlap issues are noted in the LaTeX output and references section).**

Thank you for your kind comment. The final layout was revised to fulfill PLOS ONE norms on figures, tables, and numbering. Particularly, we introduced several pagebreaks in Latex to avoid overlapping between bullets, tables, figures, and text. Moreover, the next particular changes were performed:

Figures were modified in the following aspects:

- Figures 1 through 13 incorporated a revised boldfaced title.
- Figures 1 through 13 incorporated a short description.
- Figures 5 through 13 were moved next to the first time they are called in the text.
- Added cross-references within the text for figures 5-10

Tables were modified as follows:

- Table 1. Table notes added.
- Table 2. Added the Table notes legend.
- Table 3. Added Table notes and removed parts of equations to make formulas fit.
- Table 4. Note modified.
- Table 6. Note modified.

Furthermore, to correct the specific issue mentioned about the overlapping with references, at the end of the document, we inserted page breaks to separate the final sections (Supporting information and Acknowledgements) from the Appendix, and from References in lines 1215 onwards. The result is a now a non-overlapping document.

- **Check internal consistency of line/figure/table cross‑references after typesetting and remove any residual references to earlier versions of the manuscript (e.g., “first version,” “this new version”).**

Thank you very much for this kind comment. We didn´t notice before but there where many Tables and Figures that were not cross-referenced. Furthermore, some of those that were referenced, the cross-reference was placed improperly in the sense that, it was more than two paragraphs away from either the Figure or Table that was being called. All of these errors are now corrected in the final layout. Specifically, we added crossreference for figures and Tables 5-onwards.
